# Investigation on Mechanical Properties of Functional Graded Hybrid TPMS Structures Inspired Bone Scaffolds

**DOI:** 10.3390/polym18020236

**Published:** 2026-01-16

**Authors:** İsmail Aykut Karamanli

**Affiliations:** Department of Sorgun Vocational School, Yozgat Bozok University, 66700 Yozgat, Turkey; i.aykut.karamanli@bozok.edu.tr

**Keywords:** additive manufacturing (UN SDG 9), mechanical properties, hybrid TPMS structures, functional grading, Taguchi analyses, bone scaffold surrogates (UN SDG 3)

## Abstract

Triply Periodic Minimal Surface (TPMS) structures, with their zero average curvature, excellent energy absorption properties, high specific strength and high surface-to-volume ratio, could be used in a wide range of applications, such as the creation of lightweight and durable structures, grafts and implants. In this study, an internal TPMS structure inspiring trabecular bone and an external TPMS structure inspiring cortical bone were combined with infill density and topologically functionally graded to obtain hybrid structures. The aim of the study was to investigate the effects of functional grading on mechanical properties, energy absorption capacity and surface/volume (S/V) ratio and to determine the best combination. The novelty of the study is to obtain hybrid structures close to bone structures with a functional grading approach. The experimental design of the study was performed using the Design of Experiment (DoE) approach and the Taguchi method. Specimens were created according to the established experimental design and fabricated using a Masked Stereolithography (mSLA)-type 3D printer with bio-resin. The fabricated structures were subjected to compression tests; the results were examined in terms of deformation behavior, first peak, maximum force, energy absorption, specific energy absorption and S/V ratio. The optimal structures for defined input parameters were determined using signal-to-noise (S/N) ratios and ANOVA results. Deformations for diamond and primitive specimens began as shear band formation. Deformations for Neovius structures were mostly as brittle fracture. The highest first peak of 18.96 kN was obtained with the DN specimens, while the highest maximum force of 23.77 kN was obtained with the ND specimens. The best energy absorption property was also obtained with ND. The highest S/V ratio was 5.65 in the GP specimens. The statistical analyses were in accordance with the experimental results. Infill density increases decreased the S/V ratio while increasing all other parameters. This demonstrated the importance of mechanical-strength/porosity optimization for bone scaffold surrogate applications in this study.

## 1. Introduction

The use of grafts and implants is quite common in cases of severe bone tissue loss [1]. The bone scaffolds and bone grafts used should have a porosity that allows for cell and fluid passage and permits blood vessel [1,2,3]. The material used for these applications must be biocompatible and biodegradable to avoid toxic effects on metabolism [4,5]. Currently, alloplastic (synthetic) implants and grafts made from ceramic-based materials such as hydroxyapatite (HA), tricalcium phosphate (β-TCP), bioactive glasses and calcium sulfate are generally preferred in surgical applications [6,7]. In recent years, biodegradable polymers and composites such as polylactic acid (PLA), polyglycolic acid (PGA), polylactide (PCL) and their copolymers have increasingly replaced traditional ceramic-based materials due to their controlled biodegradation behavior, elimination of secondary removal surgery and suitability for bioactive molecule incorporation during bone regeneration [8,9,10,11,12,13,14,15,16].

With the increasing use of additive manufacturing and rapid prototyping technologies in this field, developments have also been experienced in the design and production of biomedical applications such as bone implants and grafts. The use of additive manufacturing in bone implant and graft production enables customized designs that provide sensitive control over density and morphology [17], improves anatomical compatibility, reduces surgery duration and enhances biological performance in terms of cell migration, nutrient diffusion and vascularization [1,18,19,20,21,22]. Investigations into the usability of structures produced by additive manufacturing as bone implants and grafts have increased in recent years. Triply Periodic Minimal Surface (TPMS) lattices are gaining increasing attention in biomedical applications due to their continuous minimal surface geometry, the possibility of optimizing porosity–density relationships and their controllable mechanical behavior, depending on both their porosity and topology [16,23,24,25].

For a scaffold to become anatomically compatible, it is important that it resembles the nature of the area where it is applied [26,27]. Examination of the bone structure reveals that the external part consists of cortical bone, which is harder and less porous, while the internal part consists of trabecular bone, which is softer and more porous [28,29]. Comprehensive studies on polymer-based bone scaffolds fabricated by 3D printing emphasize the need to establish a balance between biocompatibility, controllable biological degradation, mechanical integrity and vascularization, as well as interconnected porous microstructures that support stress transfer for successful bone regeneration [30]. In this context, functionally graded scaffold designs are increasingly proposed to mitigate stress shielding effects and better inspire the hierarchical structure of natural bone tissue [31]. Functional grading investigations have been conducted to increase the biocompatibility between the bone region and the implant. In the research performed by Viet et al., structural functional grading was created by interlocking different TPMS structures and bone structure was inspired. Compression tests revealed that the created scaffold implants resembled bone structure [28]. In another study, functional grading was applied by changing the local densities of the specimens, and the deformation mechanisms under compressive loading were investigated [32]. Recently, hierarchical porous scaffold designs have also become possible as an alternative to scaffold implant design with density and structural functional grading. These structures enable the achievement of multi-level internal structural characteristics [33,34].

The development of additive manufacturing methods has led to an increase in studies aimed at optimizing the mechanical and morphological properties of TPMS structures used in biomedical applications. In the study examining the mechanical and energy absorption properties of biomimetic-inspired TPMS under compressive load, the TPMS were optimized structurally [35]. In another study, the effect of fabrication parameters on the tensile strength of TPMSs with different geometries produced using 3D printing was investigated, and the parameters were optimized using the Taguchi method [36]. Temiz et al. investigated the mechanical behavior of functionally graded TPMS structures with different geometries under compressive loads and optimized them. They found that the increase in density improved the mechanical properties and that diamond structures in particular exhibited superior compressive strength performance [37].

Investigation on the TPMS bone scaffold observed that studies focused on topological functional grading [38] and functional grading based on porosity variation [39]. However, neither of these two functional grading types can completely inspire trabecular and cortical bone structure. In this study, hybrid specimens were produced using additive manufacturing from biocompatible materials, to which a functional grading was applied in terms of density. The mechanical properties, failure modes and energy absorption capacities of these specimens under compressive loads were investigated. The optimization of the data obtained was performed using the Taguchi methodology.

This study investigates whether the topological and mechanical properties of functionally graded hybrid TPMS structures are suitable for use as bone scaffold surrogates. The novelty of this study differs from previous research by offering a hybrid design methodology that not only evaluates density grading or topological variations but rather evaluates these two parameters together. The study focused on establishing a bone surrogate model that combines a low-density TPMS structure, which inspires the high porosity of trabecular bone in the internal region, with a high-density TPMS structure that assumes the primary load-bearing function of cortical bone in the external region. Additionally, the surface/volume (S/V) ratios of bone scaffold surrogates were optimized through a combination of structures with different topologies. This dual-grading strategy, with the surrogates’ high S/V ratio and adjustable porosity, has the potential to provide the required conditions for vascularization. The statistical optimization of TPMS structures and density combinations using the Taguchi method and ANOVA aimed to determine the most appropriate configurations for bone surrogates in terms of mechanics. The study discussed potential implantation regions for combinations of specimens with different S/V ratios and mechanical properties and offered recommendations for in vivo studies.

## 2. Materials and Methods

### 2.1. Design and Specimen Fabrication

In this study, bone structures were inspired by interweaving two diferent TPMS structures. There are four different TPMS structures used: diamond, Neovius, gyroid and primitive. In accordance with the nature of the bone structure [28], the internal structures were designed and fabricated to have lower density (10% and 30% infill density), while the external structures were designed and fabricated to have higher density (50% and 75% infill density). The porosity of cortical bone generally ranges from 5% to 30% while that of trabecular bone can reach 50% to 90% [31]. Infill density values are selected to represent the porosity ranges of bone tissue while also allowing for the identification of differences in mechanical behavior between them, based on structural differences. The designs were generated using ANSYS SpaceClaim 2024-R2. The structures consist of cubic cells with an edge length of 1.75 mm, each oriented in the x, y and z directions. The specimens are in cylindrical shape, with an internal diameter of 20 mm, an external diameter of 30 mm and a specimen length of 60 mm in accordance with ASTM D1621-16 [40]. The designs were saved in STL format and sliced using ELEGOO SatelLite V1.0.1.29 software. The specimens were produced using an ELEGOO Saturn-16k (Shenzhen, China), a Masked Stereolithography (mSLA) type 3D printer. The fabrication parameters are a two-minute exposure and a 0.05 mm layer height. Brand bio resin (Anycubic, Shenzhen, China) which is biodegradable, was used in the fabrication. The chemical composition and mechanical properties of bio resin [41] are given in Table 1. The biodegradability of bio-resins is based on the ester bonds of the 2-oxepanone homopolymer and poly(ethylene glycol) diacrylate (PEGDA) derivatives contained in their structure [42]. Browning et al. state that the basic degradation mechanisms of this type of photopolymer structure in the in vivo environment could meet the essential requirements for biomedical applications [42]. Mondal et al. stated that composite bio-resins containing PEGDA, fabricated using the mSLA technique and used as graft material for bone repair, allowed osteoblast production in an incubated body fluid environment. They also noted that these types of composites have excellent bone bioactivity and bone defect repair potential [43]. Although vivo studies indicate that bio resins are bio-degradable and biocompatible, this study focuses on the mechanical properties of the fabricated TPMS structures and presents a mechanical surrogate analysis. It is a limitation of the study that the structures could not be examined in vivo.

**Figure 1:**
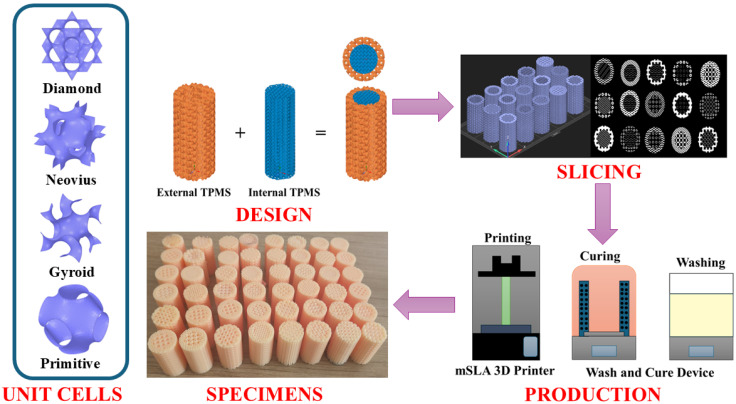
Overview of the research workflow: unit cells, design-production processes and produced specimens.

Washing and curing processes were performed using the ELEGOO Mercury Plus 3.0 device. Each specimen was washed for three minutes with 99.9% pure isopropyl alcohol and cured under UV light for 30 min. The unit cell types, design-production processes and fabricated specimens are presented in Figure 1.

### 2.2. Compression Tests

The specimens were created using the Design of Experiment (DoE) method, which yielded 16 different specimens. Compression tests on the specimens were performed using a compression test machine (Zwick/Roell, Shanghai, China) capable of 600 kN. The test speed was performed at 6 mm/minute, which is 10% of the specimen height, in accordance with ASTM D1621-16 [34,40]. The loads gradually increased until the punch reached 75% of the specimen (45 mm compression). To reduce errors in the results and increase accuracy, at least three successful tests were performed for each specimen type, and the results were averaged. Force-displacement curves were obtained through tests. The mechanical strength properties, energy absorption capacities and failure modes were analyzed using the data obtained from these curves. In addition, for the analysis of failure modes, each specimen was photographed with a DSLR camera at compression lengths of 15 mm, 30 mm and 45 mm.

### 2.3. Statistical Methods and Optimization

The optimization process for the obtained data in the study was performed using Minitab 22.4.0 software with Taguchi. The L16 mixed-type orthogonal array was used in the study. The design matrix generated is given in Table 2. The Taguchi method was used to find the best TPMS type–infill density combination. This allows reliable results to be obtained with less tests. The significance and effect ratios of the output parameters investigated were calculated using ANOVA. The confidence level for all parameters is 95% (*p* < 0.05).

The output parameters for this study are first peak, maximum force, energy absorption and specific energy absorption. In calculating the signal-to-noise (S/N) ratio, the “larger is better” method was selected to improve the strength properties of the structures, increase their energy absorption capacity and surface/volume ratio. The relevant formula is given in Equation (1).

(1) $$S/N=-10\log\left[\frac{1}{n}\left(\sum \frac{1}{y^{2}}\right)\right]$$

where *y* represents the responses for a specific factor level combination, and *n* indicates the number of responses in that combination.

### 2.4. Energy Absorption Calculations

Energy absorption (EA) denotes the cumulative energy absorbed by a structure during deformation due to external loads, typically quantified as the area under the force-displacement curves. This parameter is crucial for ductile behavior and energy absorption of the structure, particularly in applications necessitating collision and impact resistance [44,45]. Moreover, the EA value evaluates both the load-bearing capacity of the structure and its resistance to sudden fracture. Energy absorption transpires during both the elastic and plateau phases, with the majority occurring in the plateau area [46]. The equation for EA calculation is specified in Equation (2). Where $\varepsilon_{d}$ represents the displacement value at which the plateau region ends and the densification region begins. In the study, the densification phase was determined by evaluating the stress–strain curves separately for each specimen. It was assumed that the specimens entered the densification phase when the curve increased suddenly and rapidly.

(2) $$EA=\int_{0}^{\varepsilon_{d}}\sigma (\varepsilon )d\varepsilon$$

Specific energy absorption (SEA) is defined as the quotient of energy absorption and the mass of the structure (m). This measure is a vital optimization criterion, particularly in applications such as biomedical implants, automotive components and aviation constructions, where lightweight and strength are simultaneously required [47,48]. A high SEA value indicates an enhanced capacity to absorb greater energy with reduced material usage. This enhances material efficiency and promotes environmental sustainability [49,50]. The SEA calculation formula is presented in Equation (3). SAE was calculated using the total mass of the cylindrical specimen.

(3) $$SAE=\frac{EA}{m}=\frac{1}{m}\int_{0}^{\varepsilon_{d}}\sigma (\varepsilon )d\varepsilon$$

## 3. Results and Discussion

In this section, the failure modes under compressive force, compressive strengths, energy absorption capacities and S/V ratios of functional graded hybrid TPMSs were analyzed. The optimal functional grading hybrid combination for defined input parameters were determined using the Taguchi method.

### 3.1. Deformation Analysis

**Figure 2:**
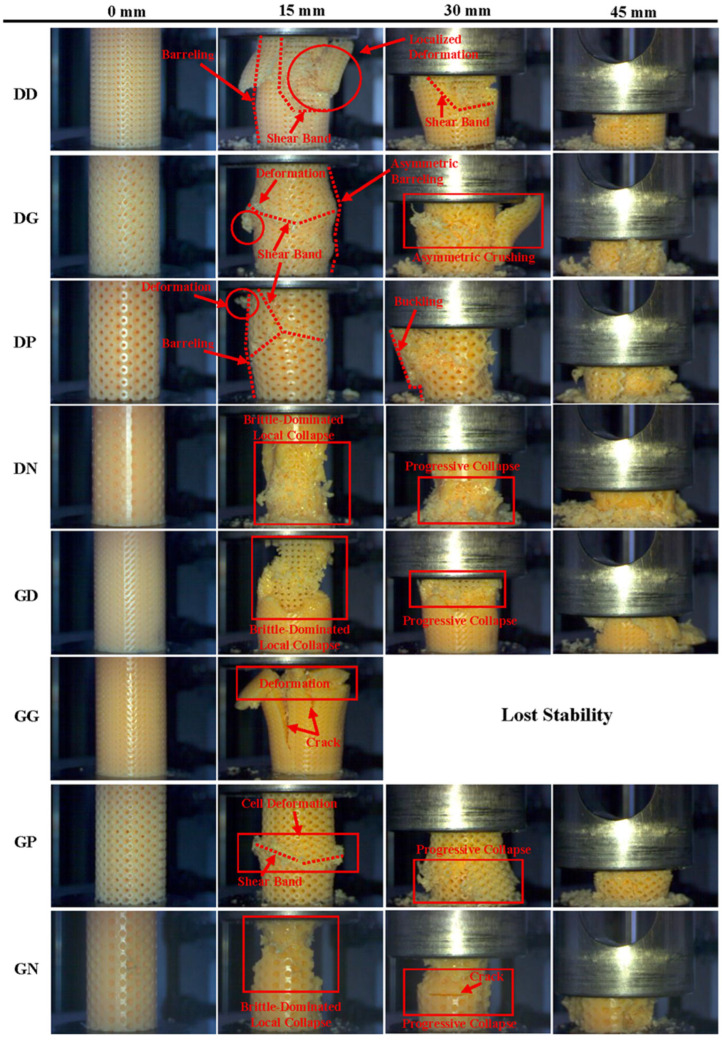
Deformation behavior of diamond and gyroid internal structure specimens at different compression levels.

**Figure 3:**
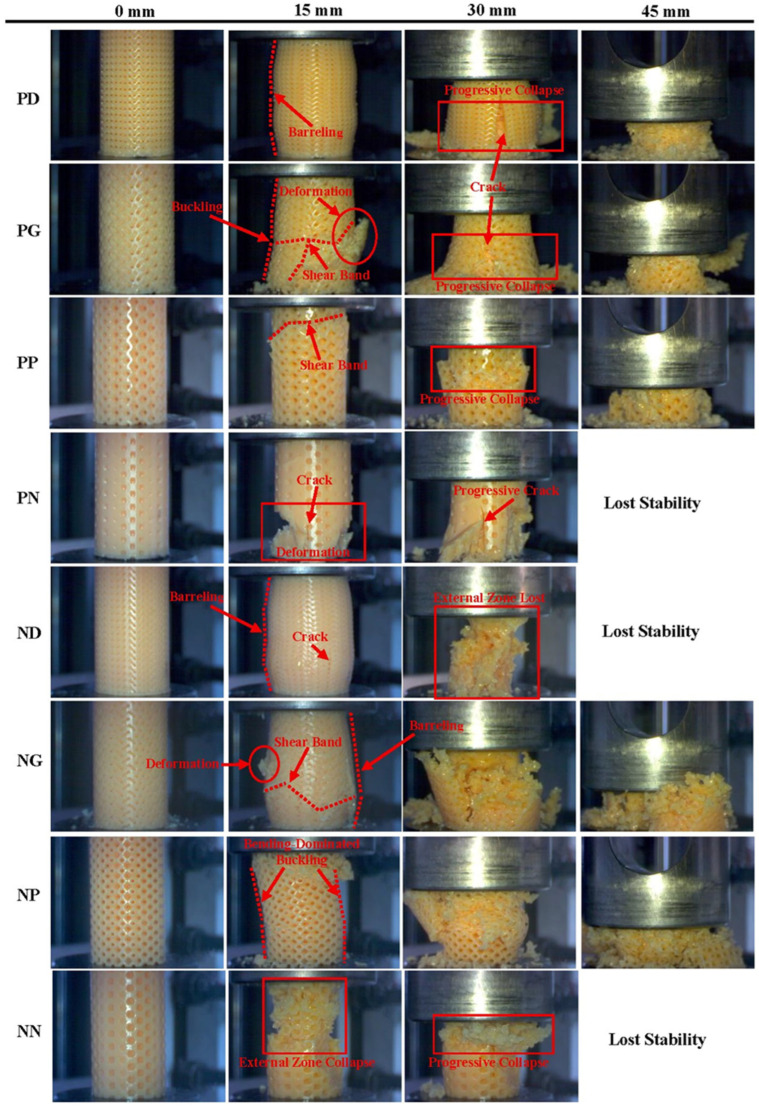
Deformation behavior of primitive and Neovius internal structure specimens at different compression levels.

Deformation modes were evaluated for all specimens by examining instantaneous images and force-displacement curves. If sudden separations occurred in the specimen structures and force values dropped suddenly by more than 30%, the deformation modes were evaluated as brittle. Failure modes for specimens with diamond and gyroid internal structures are shown in Figure 2, while failure modes for specimens with primitive and Neovius internal structures are shown in Figure 3. In all specimens with diamond internal structures except DN, barreling and shear band formation were evident at 15 mm compression. Deformations began as cell migrations around the shear band and continued to spread from around the shear band. In the DD specimens, deformations were in the form of progressive collapse. At 30 mm compression, a unilateral bending tendency was observed in the specimen due to the shear band effect. For 45 mm compression, the cells were compressed in a brittle fracture mode. In DG, unlike the other specimens, asymmetric barreling was observed at 15 mm compression. As the compression amount increased, asymmetric barreling transformed into asymmetric crushing. In DP, a shear band formation beginning at three points and converging in the middle region of the specimens was noteworthy. Buckling-type deformations were observed because of this formation. In DN, deformations began at the external sections; for 15 mm compression, brittle-dominated local collapse-type deformations formed in the Neovius cells, while the internal diamond structure maintained its stability. As the compression ratio increased, deformations in the internal diamond structure also increased, leading to progressive collapse. The barreling observed in diamond specimens could be attributed to the structure topology and the weak connections between layers resulting from this topology [29,51,52]. Shear band formation may occur due to the anisotropic effect created by cell migration [29,52].

Examination of gyroid internal specimens revealed that at 15 mm compression, diamond external cells exhibited brittle behavior, while gyroid internal cells exhibited more ductile behavior. Brittle-dominated local collapse deformation mode was dominant in the diamond external section. Deformations and collapses began in the upper parts of the specimens and gradually progressed toward the lower regions. In GG, under compressive stress, fractures first occurred in the cell supports, and as these fractures merged, cracks began to form in the external sections. At 15 mm compression, cracks developed, fissures formed and the external structure separated from the internal structure. Due to the separation effect, the specimens lost their load-bearing capacity and stability. In the deformation modes of the GP specimens, shear band formation associated with cell migration was dominant. Similarly, deformations continued to increase around the shear band and caused progressive collapse. In GN, brittle-dominated deformation was observed, as in DN. It was noteworthy that brittle fracture was generally observed in specimens with a Neovius external zone. This situation could be explained by the geometric configuration of the Neovius structures. In Neovius TPMS, the struts are highly curved and connected to each other from four different directions by load-bearing paths. This causes the perpendicular compressive loads to be distributed to the side areas as well. This may have caused the loads to affect the cells from all directions, leading to the formation of stress accumulation areas in the structure and brittle fractures. Similarly, Kumar et al. have also stated that brittle failure is observed in Neovius structures [53]. Poltue et al. found that stress accumulation in Neovius TPMS occurs in the external regions [54], which may cause brittle fracture of the external region in the Neovius structure for DN and GN specimens. Another indicator of stress accumulation is the formation of deep cracks in the external structure under 30 mm compression.

Deformations in PD specimens began with cellular bending and the resulting formation of barreling. The formation of barreling was specific, as observed in other diamond-structured specimens [51,52]. As the compression increased, the cell deformations in the external structure were transformed into cracks and fissures. The deformations continued in the form of progressive collapse. In PG, the cell deformations resulted in the formation of shear bands in three separate regions. As the compression increased, buckling occurred in the structure, the shear bands merged and crack formation ensued. Similarly, shear band formation in PP led to progressive collapse. In the PN with Neovius external structure, a more brittle deformation mode dominated, similar to DN. Cracks formed in the external region under the effect of compressive forces and brittle deformation occurred as the cracks expanded. As a result of strong deformations, it became unable to carry loads and lost its stability. Guo et al. stated that plastic hinges formed in cells because of compressive loads in primitive structures, that plastic hinges created structural orientations and that they caused buckling. The buckling that occurs in structures is the main cause of shear band formation [55]. In another study, supporting the results, it was found that the cellular buckling observed in primitive specimens caused shear forces and shear band formation [56].

The deformation behavior of Neovius internal structured specimens was examined; barrel deformation was observed in diamond and gyroid external structured specimens. In the ND specimens, the orientations in the cell walls caused crack formation, while in the NG specimens, these orientations were the primary cause of shear band formation. External cracks formed on the external regions of the ND specimens caused the external structure to collapse, while the internal structure continued to bear the load. At 45 mm compression, the internal structure also lost its stability and the structure collapsed completely. At NG, increased compression caused the shear bands in the external structure to be replaced by fractures. The fractures caused the external structure to collapse earlier. Bending-dominated buckling was predominant in the NP specimens. This situation again could be explained by the formation of plastic hedges by hollow primitive structures and the creation of structural orientations [55,56]. NN specimens exhibited more brittle deformations. At 15 mm compression, the external zone collapsed, followed by the internal zone, leading to progressive collapse. The structure completely disintegrated as the compression increased further. Stress accumulations in Neovius structures are not homogeneous; they are concentrated in cell joint regions [53,54]. This also causes structural damage and brittle fracture behavior in the cells.

Analysis of the specimens in terms of infill density revealed that as the infill densities increased, the fracture behavior generally evolved into brittle deformation. As the cell walls are thinner in structures with low infill density, strut bending, pore collapse and local buckling occur in individual cells under compression loads. These mechanisms dissipate energy, giving the structure a certain degree of ductility and energy absorption capacity [57]. However, when the stress increases, the cell walls thicken, the ability to deform decreases and the load is carried directly along the wall. This situation could lead to stress concentrating in certain areas and the formation of crack initiation zones [58].

### 3.2. Compression Test Results

The results of the compression tests are presented in Table 3. Determination strength properties of structures under compressive forces, the first peak [34,59], which represents the critical force causing the transition from elastic to plastic deformation, and the maximum force value, which represents ultimate strength, are crucial. The results indicated that both the internal and external TPMS topology and infill density have a decisive role on the first peak and maximum force. Neovius internal structures stand out with high first peak and maximum force values, while primitive and low-density diamond internal structures exhibit fracture behavior at low compressive forces. This situation is closely related to geometric topology [60,61]. For the same infill density, Neovius structures featured thicker, directly connected struts. As a result, they demonstrated an effect that increased the first peak and maximum force values [54].

Examining diamond-internal specimens reveals that the first peak values of DD and DG structures with infill density remain at the 5–5.5 kN level and the maximum force increases slightly. Due to the thin cell walls [62], the elastic region was short-standing in these specimens, and as their deformation behavior indicates, stress could have concentrated in certain areas and led to sudden fractures. In DP specimens, increasing the internal infill density ratio to 30% resulted in an increase in the first peak force, demonstrating that the infill density ratio has a direct effect on cell wall thickness and load-bearing pathways [62,63]. The DN specimens reached the highest value among all specimens with a first peak of 18.96 kN. The Neovius zone significantly increased the first peak value by directly transferring the external structure load. However, this structure did not show a significant increase in load after the first peak. This situation could be due to the limited load sharing after densification, resulting from the high rigidity of the external Neovius structure [21,61]. Gyroid internal structural specimens, particularly GD and GG, increased between the first peak and maximum force values, indicating an increase in load-bearing capacity in the densification zone of the structures. Gyroid structures allow stress redistribution as the load changes direction, increasing the load-bearing capacity of the external structure. This behavior is consistent with the distributed stress field characteristics of gyroid structures reported by Feng et al. [64]. However, the GP specimens collapsed due to the empty primitive and external structure [29,34], and no increase in force was observed after the first peak.

For specimens with a primitive internal structure, the first peak values generally remained at a mid-level; however, variations emerged in some combinations depending on the type of external structure. In the PD specimens, an increase in force was achieved after the first peak, indicating the presence of a densification zone in the structure and that the structure continued to bear the load [65,66]. For PP, both the first peak and maximum force remained at 5.99 kN. In contrast, PN reached a high first peak force of 18.20 kN, which is also the maximum force. This situation indicates that the Neovius external structure limits ductility and experiences brittle fractures, which are consistent with its deformation behavior. Neovius internal structural specimens demonstrated the highest performance among all specimens in terms of both first peak and maximum force. ND specimens in particular stood out with a first peak of 15.39 kN and maximum force of 23.77 kN. It was noteworthy that the best strength values for both the first peak and maximum force were obtained with the diamond–Neovius combination. Neovius internal structural specimens demonstrated the best performance among all specimens in terms of both the first peak and maximum force. It could be explained by the combination of the Neovius internal structure [54], which can transfer stress directly, and the diamond external structures [67], which has an excellent stress distribution due to its topology that allows for continuous repeating connections, sharing the load together. However, the rigid external structure increased deformations in the shell region and caused a loss of stability as compression increased, whereas NG demonstrated satisfactory strength performance with a first peak of 11.59 kN and a maximum force of 15.69 kN. Another important result for these specimens was that they continued to carry loads at high compressions despite their rigid Neovius internal structure. This situation could be due to gyroid structures preventing stress accumulation by forming continuously variable connections [52,68]. NP performed poorly at first peak and maximum force due to its low infill density and topological disadvantages This result could be influenced by the primitive structure’s ability to connect fewer regions [34] and its deformation behavior, which generally takes the form of sudden collapse formations [52]. For NN specimens, force increases remained limited due to the effect of brittle deformations observed in both the internal and external structures. This situation is also supported by the loss of load-bearing and stability properties of the specimens after 30 mm compression.

**Figure 4:**
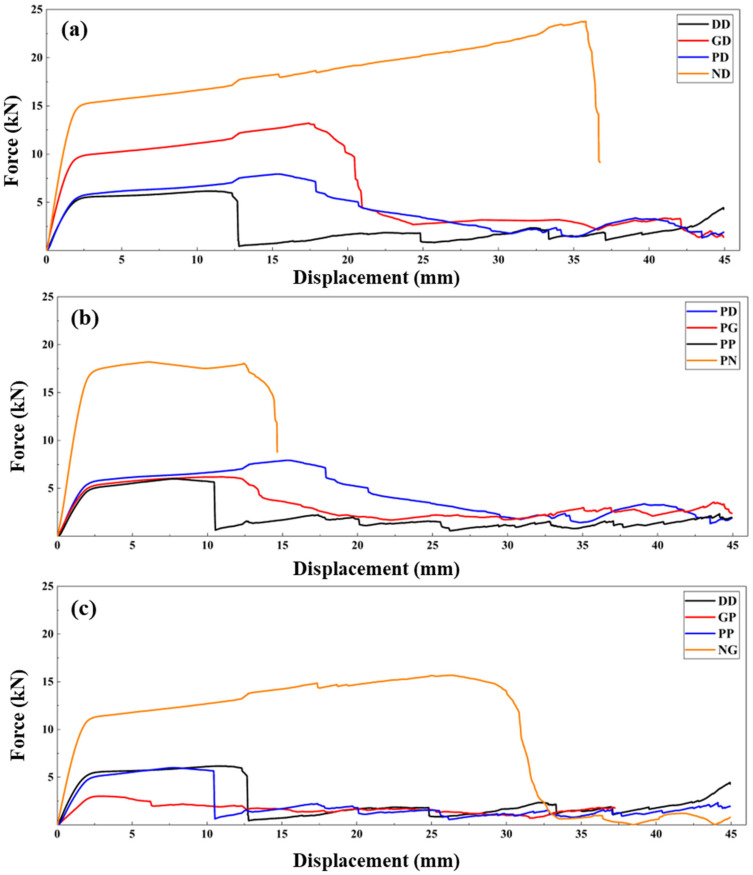
Force-displacement curves for (**a**) diamond external structures, (**b**) primitive internal structures and (**c**) different infill rates.

Figure 4 illustrates force-displacement curves for different TPMS combinations. According to Figure 4a, which compares different internal structure topologies, ND specimens achieved the first peak at a high strength value due to their high rigidity. Subsequently, it exhibited a steadily increasing plateau behavior. This is an indication of its high energy absorption capacity [45]. However, due to its rigid Neovius internal structure, it lost stability before the compression process was completed. The curves for GD and PD were similar, but the GD specimens had a longer plateau phase [52,68] due to the gyroid internal structure’s ability to excellent stress distribution. Whereas DD exhibited lower strength and a plateau region due to the effects of deformation behavior. Figure 4b compares different external structure topologies. Due to its high rigidity, PN reached a very high first peak. However, it lost its stability and disintegrated at approximately 15 mm of compression. PD and PG, on the other hand, had a more stable plateau region. The load carried in the plateau region gradually decreased because of the collapsing primitive internal structure. In PP, after reaching first peak similar to PD and PG, a sudden collapse effect occurred. Although the structure continued to bear load afterwards, it could not maintain stability. Gradual collapses caused peak formations [52,69]. Force-displacement curves for specimens with different infill densities are presented in Figure 4c. NG exhibited high first peak and maximum force values. The steadily increasing curve in the plateau region indicates high energy absorption and stable deformation progression. Increased energy absorption and more stable deformations are the result of high infill densities (30% for the internal structure and 75% for the external structure). As the infill density increases, the thickness of the cell walls also increases, which leads to increased cell rigidity [70]. Increased rigidity also has a positive effect on compressive strength. However, this rigidity gain may also cause brittle deformations [71]. After 30 mm compression, NG was subjected to brittle deformation and its load-bearing capacity rapidly decreased. The compressive strength of the PP specimens with the same external infill density as NG were quite low. The reason for this, as mentioned previously, was the internal structure at 10% infill density, in addition to the topological configuration effect. Similarly, despite DD’s topological advantage, the reason it has a lower maximum force compared to PP is that it has a lower external infill density (50%). Likewise, GP, which has the same internal infill density as NG, exhibited quite low strength properties due to similar effects.

Although this study is a mechanical surrogate analysis, the mechanical similarity of the specimens to bone structure was not ignored. One indicator of a structure’s suitability for use as a bone scaffold is its mechanical compatibility [72]. Previous studies have indicated that scaffolds or implants that are mechanically different from bone structure may damage surrounding tissues and, due to differences in load bearing, may cause decreases in bone density and bone resorption [72,73]. Supporting these results, Temiz et al. [74], applied compression tests to sheep femur bone with an average diameter of 25 mm, which is reported to have properties quite close to human bone [75,76] and found the maximum force value of the bone to be approximately 18 kN. This indicates that DN, PN and ND may be potential designs with mechanical compatibility, especially considering that the diameter of the specimens (30 mm) investigated in the study are slightly larger.

### 3.3. Energy Absorption Capacity and Surface/Volume Ratio Results

**Figure 5:**
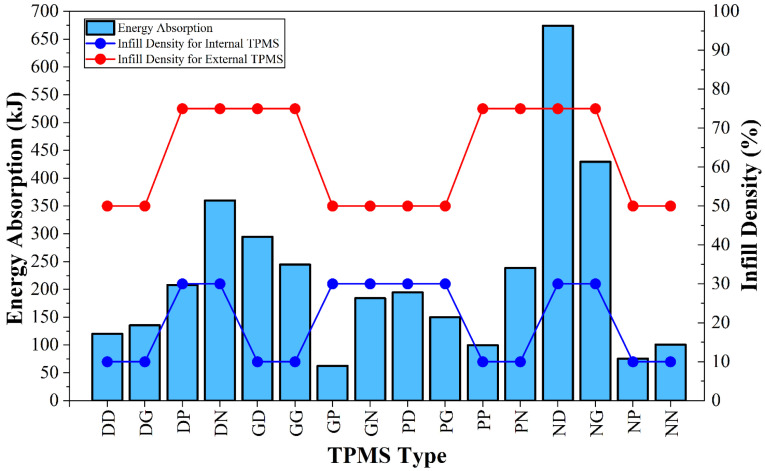
Changes in energy absorptions according to infill density and TPMS topology.

Energy absorption in TPMS structures is an indicator of impact resistance [77] and deformation stability [78]. Energy absorption results are presented in Figure 5. EA is significantly affected by both topological differences and changes in infill density. The EA capacities of the specimens also increased with increasing infill density. The ND specimen with 30% internal density and 75% external density performed most efficiently, exhibiting an EA value exceeding 670 kJ. As infill density increases, cell wall thicknesses also increase [62], which extends deformation periods and enables more efficient plateau phases to be established [65,79]. Furthermore, an increase in infill density also increases the rigidity of the structure [80] and improves EA performance [53]. Results were examined in terms of specimen topology, ND and NG specimens exhibited quite good EA performance. This situation could be related to the good stress distribution and high rigidity of Neovius specimens, in parallel with the maximum force results. GP (62.82 kJ) and NP (75.90 kJ) specimens exhibited the lowest EA performance. This might indicate that the primitive external structure’s porous topology prevents homogeneous stress distribution and exhibits short plateau behavior [68,81]. Another noteworthy result was that specimens with homogeneous structures (same internal and external structures except for GG) exhibited relatively low EA performance. This highlights the importance of heterogeneous structures in applications where energy absorption capacity is critical [82].

**Figure 6:**
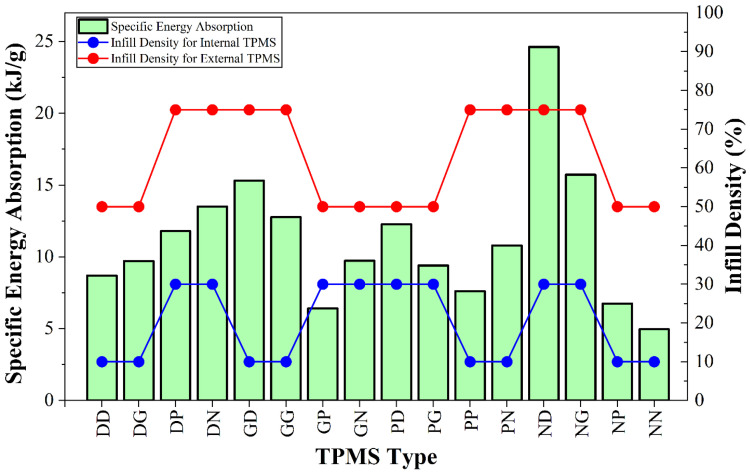
Changes in specific energy absorptions according to infill density and TPMS topology.

Specific energy absorption [83], which expresses the energy absorbed per unit mass of a structure, enables the design of high energy absorption capacity and lightweight structures [84]. The SEA values obtained from the compression tests are given in Figure 6. As the infill density increased, the SEA increased similarly to the EA. The highest SEA was 24.62 kJ/g in ND with 30% internal density and 75% external density. The lowest SEA was 4.97 kJ/g in the NN specimens with 10% internal density and 50% external density. GP and NP specimens also exhibited low performance. The reason for the improvement in SEA performance with increasing infill density is the increase in surface areas that facilitate stress distribution and the limitation of plastic deformation due to the increase in density [85]. The study, which examined the deformation behavior of diamond and gyroid TPMSs in a manner consistent with the results, stated that an increase in relative density increases SEA [64]. The reason NG and ND specimens exhibit quite good SEA performance is the combination of diamond and gyroid internal structures, which have a more ductile characteristic, with a more rigid Neovius external structural characteristic. In these combinations, the internal and external structures, which exhibit different characteristic properties, have supported each other. NN exhibited low performance due to the effects of brittle deformation. Another noteworthy result was that the other homogeneous specimens, except for GG, exhibited low SEA performance. This may also be evidence that TPMSs with different characteristics exhibit synergetic properties [82].

**Figure 7:**
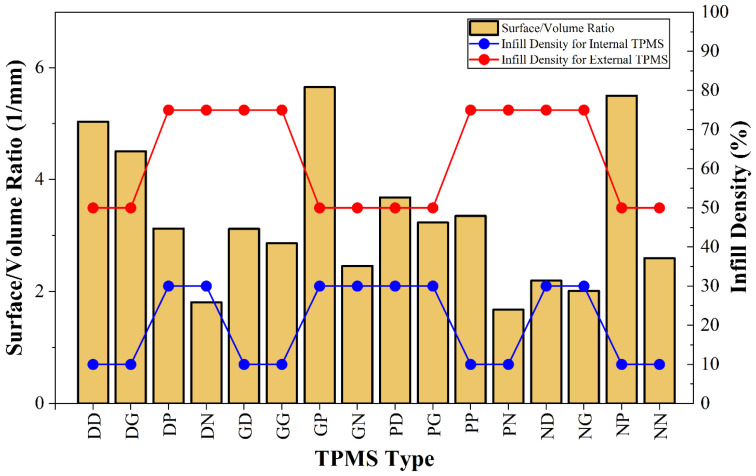
Changes in surface/volume ratios according to infill density and TPMS topology.

High S/V ratios are preferred in biomedical applications [34,53] and engineering processes requiring high levels of heat and mass transfer [86] due to their ability to enhance mass transfer and regeneration. However, increasing the S/V ratio too much reduces infill density and causes a decrease in mechanical properties and energy absorption capacity [53,61]. The S/V ratio results are shown in Figure 7. The highest S/V ratios were 5.65 1/mm in the GP specimens and 5.50 1/mm in the NP specimens. The specimens with the lowest S/V ratio were PN at 1.68 1/mm. DN also had a low S/V ratio. Changes in infill density were particularly influential on results. Wall thickness increases with increasing infill density. Therefore, this reduces porosity and consequently surface area [87,88].

Analyzing the S/V ratios for human bone revealed that, although values varied according to age, gender and the location of the bone, the results obtained were reasonable [89,90]. Lerebours et al. stated that the S/V ratio for the femoral cortex varies between 4 and 7 mm^−1^ [89]. This also indicates that DD, DG, GP and NP may be potential bone surrogates for the femoral cortex in terms of the S/V ratio. In another study, the S/V ratio was found to be 4.6 mm^−1^ for maxillary bone and 3.9 mm^−1^ for mandibular bone [90]. Therefore, DG and DP combinations could be evaluated as maxillary and mandibular bone surrogates in terms of the S/V ratio.

### 3.4. Taguchi Analysis Results

Taguchi and ANOVA analyses were used to determine the variables affecting the results and their effect ratios. The ANOVA results are presented in Table 4. Changes in external TPMS type and density had a significant effect (*p* < 0.05) on the first peak. The changes in the type and density of the internal TPMS did not have a statistically significant effect on the first peak (*p* > 0.05). This indicates that the transition from elastic deformation to plastic deformation began on the external part. The effect of the TPMS type on the first peak for the external region was calculated as 46.62%, while the effect of infill density was calculated as 42.23%. The same was true for maximum force. The effect of the TPMS type on maximum force for the external region was calculated as 38.35%, while the effect of infill density was calculated as 44.54%. Although no statistically significant effect of changes in the type and density of the internal TPMS on the first peak and maximum force was found, deformation mode analyses and energy absorption calculations show that hybrid structures exhibit synergic behavior under loading. This situation highlights the necessity of evaluating experimental data together with statistical models. For example, the results obtained indicate that Neovius structures generally exhibited higher first peak and maximum force values. In accordance with this assumption, when comparing the NN and GN specimens with the same external infill density (50%), the NN specimens with a Neovius internal structure are expected to exhibit higher force values. However, experimental results demonstrated that the GN specimens had significantly higher first peak and maximum force values when compared to the NN specimens. This could be explained by the GN specimens having a higher internal infill density (30%) than the NN specimens (10%). Although the effect of internal infill density change on the first peak and maximum force was not found to be statistically significant according to ANOVA results, it is clearly seen that it is experimentally effective in specific combinations.

All input parameters were effective on EA changes. The most effective parameter on EA changes was external TPMS density at 38.84%. All parameters except internal TPMS type were effective on SEA and S/V ratio changes.

Data for the model created using Taguchi and ANOVA are given in Table 5. The R^2^ value, which expresses the regression between the model and the results, was 88% and above. This indicates that the statistical data obtained is acceptable [91].

**Figure 8:**
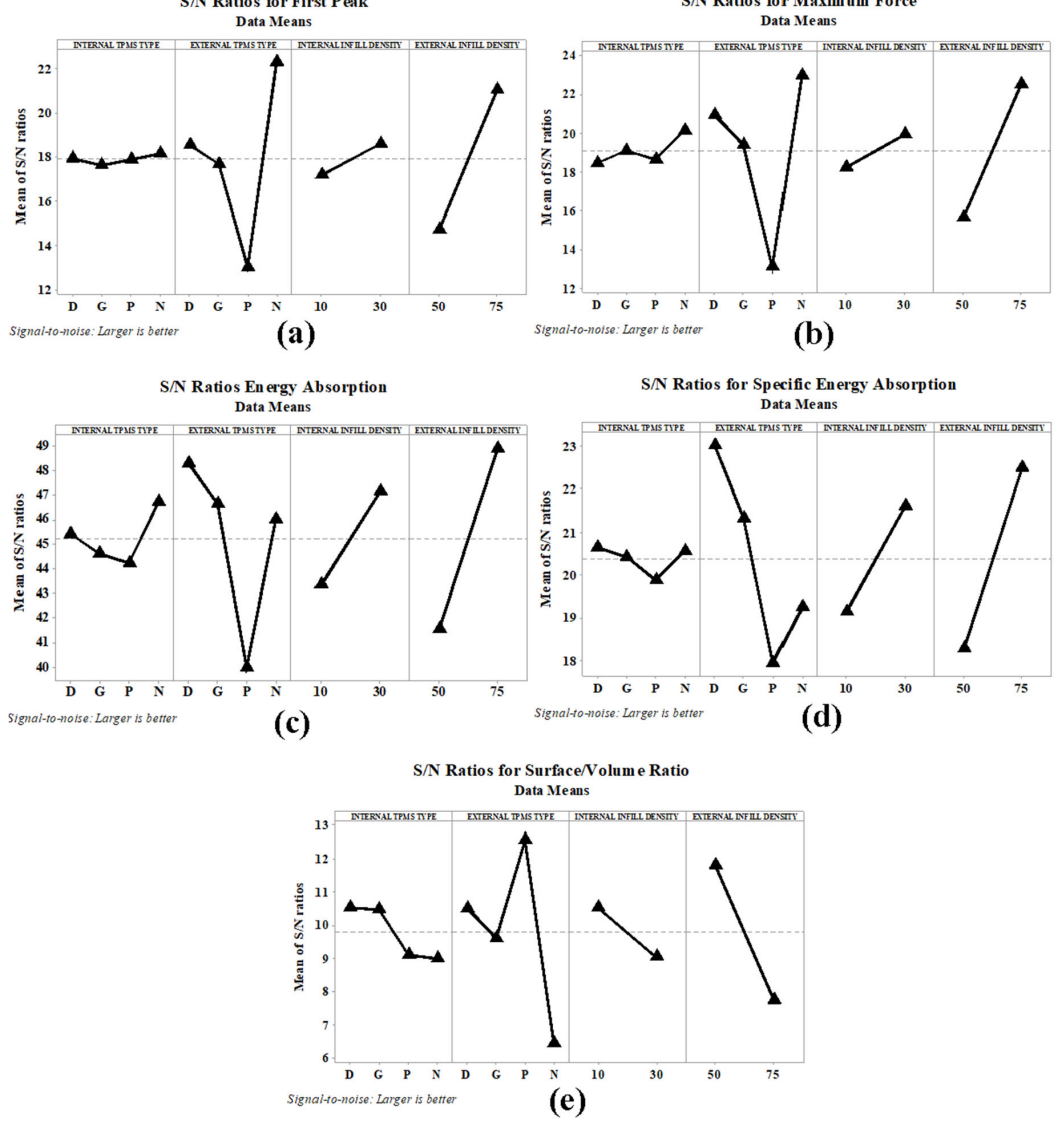
S/N ratio changes of (**a**) first peak, (**b**) maximum force, (**c**) energy absorption, (**d**) specific energy absorption, (**e**) surface/volume ratio.

The S/N ratios for the Taguchi analyses are illustrated in Figure 8. The S/N ratios for the first peak (Figure 8a) and maximum force (Figure 8b) indicated that the internal TPMS type had no significant effect. For the external TPMS type, the best results were obtained in Neovius structures, while the poorest results were obtained in primitive structures. Diamond and gyroid structures exhibited average performance. An increase in infill density positively affected the first peak and maximum force values, but the effect of the external infill density increase was greater. The best EA performance (Figure 8c) was achieved with the Neovius internal-diamond external structure combination. The gyroid external structure also had quite good EA performance. The worst performance was in the PP combination. For SEA (Figure 8d), the effect of changing the internal TPMS type was limited. In contrast, the effect of the external TPMS type was significant. Diamond and gyroid external topology structures exhibited good performance, while primitive external topology structures demonstrated low SEA performance. Increases in infill density indicated positive effects for both EA and SEA. S/N ratios (Figure 8e) were examined for S/V ratio results; diamond and gyroid internal structure specimens were found to achieve high values. The S/V ratios remained low for Neovius and primitive structures. For the external zone, primitive structures had satisfactory values, while the values for Neovius structures were quite low. Gyroid and primitive external structure specimens achieved average S/V ratios. With an increase in infill density, the S/V ratios experienced a dramatic decrease.

In this study, the DoE approach, which is based on the principle of reaching the correct result with minimal experiments, was used. To limit the experimental load while maintaining orthogonality in Taguchi experimental design, only two different levels were used for internal (10%, 30%) and external (50%, 75%) infill densities. However, considering that parameters such as force values, energy absorption properties and the S/V ratio are strongly affected by changes in infill density, evaluating infill density between two values constitutes a significant limitation of the study. There is also a possibility that the actual optimum values may occur within intermediate density ranges such as 30–50% or 50–75%. Nevertheless, as previously mentioned, infill density values were determined based on human bone structure [31], which enhances the accuracy of the selected infill density ranges and the data obtained in the study. Even so, the optimal configurations obtained as a result of Taguchi analyses should be evaluated as an optimal value obtained within defined input parameters rather than an absolute optimum. This approach is suitable for determining critical parameters, investigating the effects of factors and establishing pre-designs; however, it may not accurately represent the continuity of density–mechanical property relationships and their potential non-linear behavior, which are of critical importance in biomedical applications. Viewed from a biomedical design perspective, this limitation means that the proposed optimal configuration does not represent an absolute optimal design, but rather a range of feasible designs. Therefore, in future studies, the inclusion of intermediate infill density levels and the in vivo validation of findings with personalized biomedical applications will enable more accurate calculation of output parameters.

The results generally indicate that increasing the infill density increases strength and energy absorption capacity; however, the S/V ratio decreases. While this increase in infill density enhances the strength of bone scaffolds [28], it may also hinder vascularization, material and cell migration [89]. When examining bone tissue in different regions of the human body, in parallel with the study, it is observed that bone densities vary. The cortical bone ratio in the spinal bone is 25%, while the trabecular bone ratio is 75% [92]. For the femur, this ratio is 50-50, whereas for bones with a higher load-bearing potential, such as the forearm (radius), it is higher [93]. Furthermore, the average loads carried by the bones also differ. This also indicates that different designs may be more suitable for different regions. For bones that carry less load and low-density bones, such as the spine, specimens with 10% internal and 50% external density ratios may be preferred. DD and DG specimens may be preferable choices, especially for the design family utilized in the study. For structures with higher density, such as the femur bone, that carry heavy loads, the GN specimens with a 30% internal-50% external density ratio or the GD and GG specimens with a 10% internal-75% external density ratio may be options within this design family. For bones that may be subjected to high loads, such as the forearm, and therefore require high energy absorption capacity, ND and DN specimens with higher infill density and greater load-bearing capacity would be more appropriate choices. On the other hand, in graft applications that do not carry any loads or only carry low loads, a high S/V ratio is important [21]. NP, GP, DD and DG specimens with high S/V ratios are preferred options within this design family for applications of this type.

## 4. Conclusions

In this study, deformation behavior, compression strength and energy absorption capacity of density-graded and topologically graded TPMS structures under compressive loads, as well as changes in their surface-to-volume ratio, were investigated, particularly regarding their potential for biomedical applications. The optimum TPMS topology and infill density for defined input parameters were determined using Taguchi and ANOVA. Deformation behaviors for diamond and primitive specimens began primarily with the shear band formation. Deformations were more progressive in these specimens. More brittle deformations were dominant in Neovius structures. Brittle deformations also increased as the infill density increased in the structure. The highest first peak was obtained in the DN specimen at 18.96 kN, while the highest maximum force was obtained in the ND specimen at 23.77 kN. It is noteworthy that the best compressive strength properties are achieved with the diamond-Neovius combination. On the other hand, primitive-structured specimens generally achieved lower strength values due to their topological disadvantages.

The ND specimens performed best in energy absorption with values of 674.21 kJ EA and 24.62 kJ/g SEA. NN, NP and GP specimens exhibited the lowest SEA and EA performance. Primitive external specimens showed low energy absorption performance due to the effect of topological properties. In general, homogeneous specimens achieved lower EA and SEA values than heterogeneous ones. This may indicate that TPMSs with different characteristics could produce synergic effects. The specimens with the highest S/V ratios were GP and NP, while those with the lowest S/V ratios were PN and DN. The increase in infill density increased the first peak, maximum force, EA and SEA values but decreased the S/V ratio. The Taguchi analyses highlighted Neovius external topologies with high-density structures in the applications where strong strength properties were desired, demonstrating the necessity of optimization processes. The high-density ND hybrid structures were suitable for applications requiring high energy absorption. The results showed that hybrid structures with diamond external topologies could be preferred for lightweight applications with relatively high energy absorption capacity. In applications where surface area is critical, low-density DP and GP structures were found to be preferable.

The study found that the structures investigated were remarkably similar to bone tissue in terms of mechanical properties, energy absorption capacity and S/V ratio. Nevertheless, there are still many aspects that need to be investigated before the proposed structures can be used in biomedical applications. Although the study focused on the mechanical behavior of hybrid TPMS structures under static compressive loads, it is known that bone scaffolds are subjected to dynamic and multi-axial loading in the actual in-vivo environment. Hybrid designs, especially at the interfaces where different topologies meet, may exhibit a reduction in the fatigue life of bone scaffold surrogates due to stress concentration. In future studies, performing fatigue tests on hybrid TPMS designs and evaluating their performance under complex loading conditions is critical for enhancing the clinical feasibility of these designs. This study proposes a functional grading approach for hybrid TPMS bone scaffold surrogate applications. The main limitation of the study is that the functional grading hybrid TPMS approach has not yet been physiologically validated. Future studies aim to validate the proposed approach through animal experiments and demonstrate its potential for biomedical applications.

## Figures and Tables

**Table 1:** Chemical composition and mechanical properties of Anycubic bio resin [41].

Chemical Composition of Bio Resin	
2-Oxepanone, homopolymer, 2-[(1-oxo-2-propen-1-yl)oxy]ethyl ester	30–60%
Oxirane, 2-methyl-, polymer with oxirane, bis(2-methyl-2-propenoate), block	20–40%
1,2-Ethanediyl bisacrylate	25–40%
phenyl bis (2,4,6-trimethylbenzoyl)-phosphine oxide	2–5%
**Mechanical Properties**	
Density	1.13–1.15 kg/mm^3^
Hardness	83–85 Shore D
Tensile Strength	35–40 MPa
Elongation	21–28%

**Table 2:** Taguchi L16 orthogonal array design matrix for functional graded hybrid TPMS specimens.

Specimen Code	Internal TPMS Type	Infill Density for Internal TPMS (%)	External TPMS Type	Infill Density for External TPMS (%)
DD	Diamond	10	Diamond	50
DG	Diamond	10	Gyroid	50
DP	Diamond	30	Primitive	75
DN	Diamond	30	Neovius	75
GD	Gyroid	10	Diamond	75
GG	Gyroid	10	Gyroid	75
GP	Gyroid	30	Primitive	50
GN	Gyroid	30	Neovius	50
PD	Primitive	30	Diamond	50
PG	Primitive	30	Gyroid	50
PP	Primitive	10	Primitive	75
PN	Primitive	10	Neovius	75
ND	Neovius	30	Diamond	75
NG	Neovius	30	Gyroid	75
NP	Neovius	10	Primitive	50
NN	Neovius	10	Neovius	50

**Table 3:** Compression test results of functional graded hybrid TPMS.

Specimen Code	First Peak (kN)	Maximum Force (kN)	Energy Absorption (kJ)	Specific Energy Absorption (kJ/g)	Surface/Volume Ratio (1/mm)
DD	5.54 ± 0.32	6.17 ± 0.43	120.15 ± 11.85	8.70 ± 0.86	5.03
DG	5.17 ± 0.28	5.67 ± 0.37	135.63 ± 12.17	9.72 ± 0.87	4.51
DP	7.12 ± 0.57	7.32 ± 0.46	208.16 ± 17.89	11.80 ± 1.01	3.12
DN	18.96 ± 1.12	19.14 ± 1.23	360.13 ± 24.15	13.51 ± 0.91	1.81
GD	9.92 ± 0.83	13.22 ± 1.09	294.56 ± 23.87	15.31 ± 1.24	3.12
GG	10.03 ± 0.81	13.67 ± 0.89	244.70 ± 19.43	12.77 ± 1.01	2.86
GP	3.01 ± 0.22	3.01 ± 0.22	62.82 ± 5.62	6.41 ± 0.57	5.65
GN	11.08 ± 0.92	12.15 ± 1.01	184.11 ± 13.78	9.73 ± 0.73	2.46
PD	6.02 ± 0.58	7.94 ± 0.63	194.52 ± 9.75	12.27 ± 0.62	3.68
PG	5.73 ± 0.56	6.20 ± 0.71	150.28 ± 16.02	9.40 ± 1.00	3.23
PP	5.99 ± 0.49	5.99 ± 0.49	99.74 ± 7.93	7.61 ± 0.58	3.35
PN	18.20 ± 1.74	18.20 ± 1.19	238.61 ± 31.87	10.79 ± 1.44	1.68
ND	15.39 ± 1.17	23.77 ± 1.82	674.21 ± 46.30	24.62 ± 1.69	2.20
NG	11.59 ± 0.93	15.69 ± 1.09	429.34 ± 33.14	15.72 ± 1.21	2.01
NP	3.12 ± 0.39	3.12 ± 0.32	75.90 ± 6.92	6.75 ± 0.61	5.50
NN	7.61 ± 0.61	9.19 ± 0.74	101.09 ± 9.82	4.97 ± 0.48	2.60

**Table 4:** ANOVA results of functional graded hybrid TPMSs.

	Source	DF	Contribution (%)	F-Value	*p*-Value
First Peak (kN)	Internal TPMS Type	3	0.50	0.15	0.925
	Infill Density for Internal TPMS	1	3.01	2.75	0.141
	External TPMS Type	3	46.62	14.23	0.002
	Infill Density for External TPMS	1	42.23	38.67	0.000
	Error	7	7.64		
	Total	15	100		
Maximum Force (kN)	Internal TPMS Type	3	5.34	1.69	0.254
	Infill Density for Internal TPMS	1	4.41	4.19	0.080
	External TPMS Type	3	38.35	12.16	0.004
	Infill Density for External TPMS	1	44.54	42.38	0.000
	Error	7	7.36		
	Total	15	100		
Energy Absorption (kJ)	Internal TPMS Type	3	14.06	4.04	0.049
	Infill Density for Internal TPMS	1	15.18	13.07	0.009
	External TPMS Type	3	23.80	6.83	0.017
	Infill Density for External TPMS	1	38.84	33.46	0.001
	Error	7	8.13		
	Total	15	100		
Specific Energy Absorption (kJ/g)	Internal TPMS Type	3	5.69	1.22	0.371
	Infill Density for Internal TPMS	1	13.44	8.65	0.022
	External TPMS Type	3	33.56	7.20	0.015
	Infill Density for External TPMS	1	36.42	23.43	0.002
	Error	7	10.88		
	Total	15	100		
Surface/Volume Ratio (1/mm)	Internal TPMS Type	3	4.99	2.08	0.192
	Infill Density for Internal TPMS	1	5.20	6.50	0.038
	External TPMS Type	3	43.74	18.22	0.001
	Infill Density for External TPMS	1	40.48	50.60	0.000
	Error	7	5.60		
	Total	15	100		

**Table 5:** Model summary.

	S	R^2^ (%)	Adj. R^2^ (%)
First Peak (kN)	2.0068	92.36	83.62
Maximum Force (kN)	2.4405	92.64	84.23
Energy Absorption (kJ)	65.9120	91.87	82.59
Specific Energy Absorption (kJ/g)	2.2826	88.12	76.69
Surface/Volume Ratio (1/mm)	0.4398	94.40	88.00

## Data Availability

The data presented in this study are available on request from the corresponding author. The data are not publicly available due to privacy restrictions.
